# ICTV Virus Taxonomy Profile: *Iridoviridae*

**DOI:** 10.1099/jgv.0.000818

**Published:** 2017-05-30

**Authors:** V. Gregory Chinchar, Paul Hick, Ikbal Agah Ince, James K. Jancovich, Rachel Marschang, Qiwei Qin, Kuttichantran Subramaniam, Thomas B. Waltzek, Richard Whittington, Trevor Williams, Qi-Ya Zhang, ICTV Report Consortium

**Affiliations:** ^1^​ Department of Microbiology, University of Mississippi Medical Center, Jackson, MS 39216, USA; ^2^​ Faculty of Veterinary Science, University of Sydney, Sydney NSW, Australia; ^3^​ Department of Medical Microbiology, Acibadem Medical School, Istanbul, Turkey; ^4^​ Department of Biological Sciences, California State University, San Marcos, CA 92096, USA; ^5^​ Laboklin GmbH and Co., Bad Kissingen, Germany; ^6^​ Key Laboratory of Tropical Marine BioResources and Ecology, South China Sea Institute of Oceanology, Chinese Academy of Sciences, Guangzhou, PR China; ^7^​ Department of Infectious Diseases and Pathology, College of Veterinary Medicine, University of Florida, Gainesville, FL 32611, USA; ^8^​ Instituto de Ecologia AC, Xalapa, Veracruz 91070, Mexico; ^9^​ State Key Laboratory of Freshwater Ecology and Biotechnology, Institute of Hydrobiology, Chinese Academy of Sciences, Wuhan, Hubei, PR China

**Keywords:** *Iridoviridae*, ICTV report, taxonomy, frog virus 3, ranavirus, invertebrate iridescent virus

## Abstract

The *Iridoviridae* is a family of large, icosahedral viruses with double-stranded DNA genomes ranging in size from 103 to 220 kbp. Members of the subfamily *Alphairidovirinae* infect ectothermic vertebrates (bony fish, amphibians and reptiles), whereas members of the subfamily *Betairidovirinae* mainly infect insects and crustaceans. Infections can be either covert or patent, and in vertebrates they can lead to high levels of mortality among commercially and ecologically important fish and amphibians. This is a summary of the current International Committee on Taxonomy of Viruses (ICTV) Report on the taxonomy of the *Iridoviridae,* which is available at www.ictv.global/report/iridoviridae.

## Virion

**Fig. 1.**
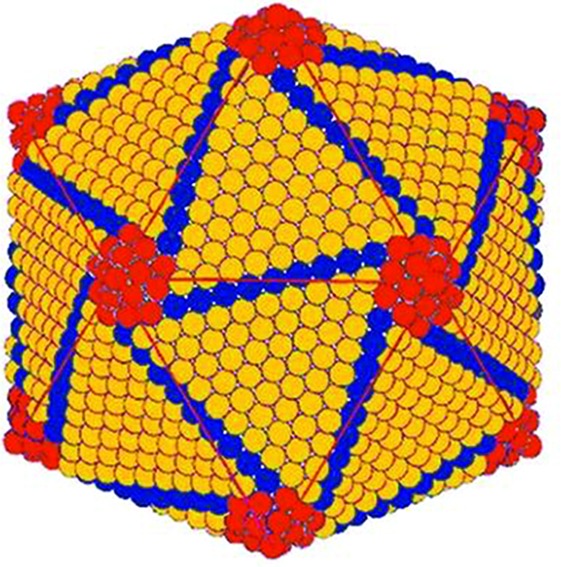
Proposed structure of the capsid of invertebrate iridescent virus 2. Trisymmetrons (orange) comprising the icosahedral faces, pentasymmetrons (red) located at the vertices, and disymmetrons (blue) at the edges of the faces are shown. (Adapted with permission from Wrigley, *J Gen Virol* 1969; 5:123–134).

Virions display icosahedral symmetry and contain an internal lipid membrane located between the DNA–protein core and the outer capsid [1] (Table 1, Fig. 1). Depending upon the genus, the capsid may have numerous external fibrils. Mature infectious virions may remain non-enveloped or bud from the plasma membrane and acquire an envelope.

**Table 1.** Characteristics of the family *Iridoviridae*

Typical member:	frog virus 3 (AY548484), species *Frog virus 3,* genus *Ranavirus*
Virion	Typically 150–200 nm (non-enveloped); the principal component of the capsid is the major capsid protein (mol wt 48 kDa)
Genome	Linear, double-stranded circularly permuted, terminally redundant DNA, 103–220 kbp, encoding 92–211 proteins
Replication	First-stage DNA synthesis and early transcription takes place in the nucleus; subsequently DNA concatemer formation and late transcription occur in the cytoplasm; virion morphogenesis takes place in cytoplasmic assembly sites
Translation	Directly from capped, non-polyadenylated mRNAs
Host Range	Amphibians, reptiles, fish (subfamily *Alphairidovirinae*); mainly insects and crustaceans (subfamily *Betairidovirinae*)
Taxonomy	Five genera divided between two subfamilies

## Genome

The virus genome is a single molecule of double-stranded DNA that is circularly permuted and terminally redundant [2]. The degree of redundancy varies from 5–50 % depending on the virus species. The genomes of all vertebrate iridoviruses, with one exception, are highly methylated due to a virus-encoded DNA cytosine methyltransferase. Twenty-six core proteins are common to all members of the family [3].

## Replication

**Fig. 2.**
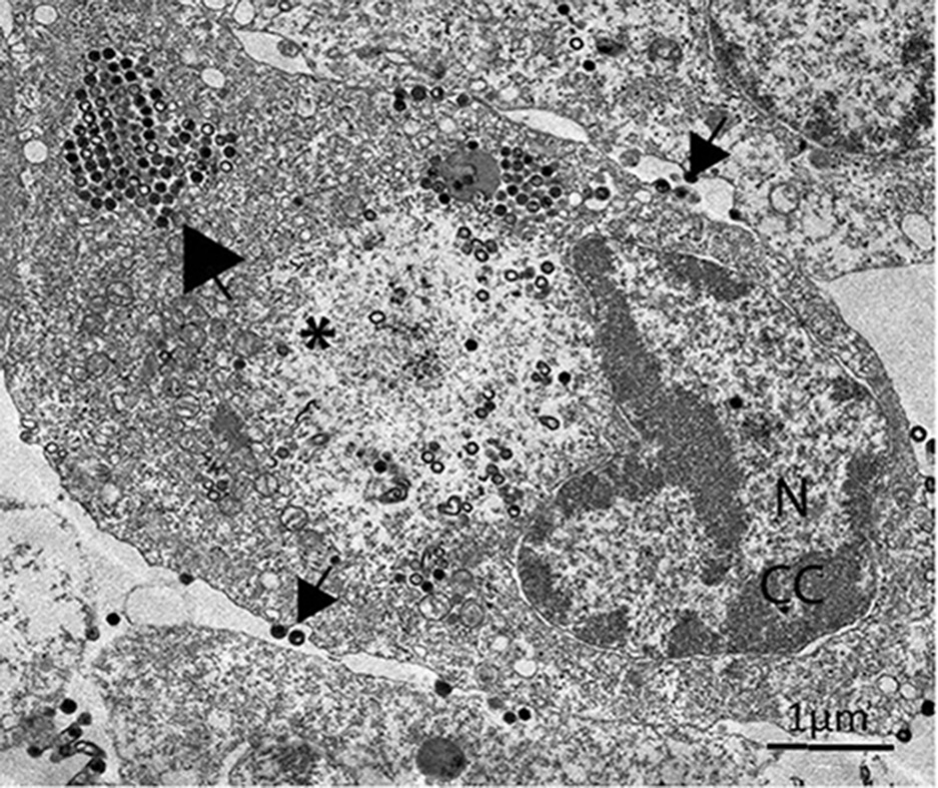
Transmission electron micrograph of a fathead minnow cell infected with frog virus 3. Large arrow, paracrystalline array; small arrows, budding virions; N, nucleus displaying condensed chromatin (CC) indicative of apoptosis; *, virus assembly site showing empty and complete virus particles; scale bar=1 µm (RC Sample and VG Chinchar, unpublished).

Replication involves both nuclear and cytoplasmic compartments [4]. In the nucleus, host RNA polymerase II directs the synthesis of immediate–early virus mRNAs. In addition a virus-encoded DNA polymerase synthesizes genome- to twice-genome-size copies of the incoming DNA. This DNA is transported to the cytoplasm, where it undergoes a second round of replication to form large concatemers. Late gene expression is catalysed by a virus-encoded transcriptase. Virion morphogenesis occurs within assembly sites, and completed particles form paracrystalline arrays or bud from the plasma membrane (Fig. 2).

## Taxonomy

Phylogenetic analyses indicate that invertebrate and vertebrate iridovirus lineages diverged early during the evolution of the family. Ascoviruses appear to have emerged recently from the former lineage and share with iridoviruses a common ancestry with marseilleviruses [5].

### *Ranavirus* (*Alphairidovirinae*)

Ranaviruses are promiscuous pathogens capable of infecting three classes of ectothermic vertebrates (bony fish, amphibians and reptiles) [6]. Ranaviruses infect not only multiple species within a class, but some (e.g. Bohle iridovirus) are also capable of infecting hosts from different classes. Infections are systemic, involve multiple internal organs and may lead to high levels of morbidity and mortality among cultured, commercially important fish and amphibians, as well as endangered wild species.

### *Megalocytivirus* (*Alphairidovirinae*)

Members infect >50 species of marine and freshwater fish; systemic disease involves multiple internal organs.

### *Lymphocystivirus* (*Alphairidovirinae*)

Lymphocystiviruses infect >100 species of marine and freshwater fish, leading to the formation of wart-like growths composed of clusters of individual, infected cells (some as large as 1 mm) primarily on the skin, but sometimes on internal organs. Morbidity may be high but mortality tends to be low.

### *Iridovirus and Chloriridovirus* (*Betairidovirinae*)

Members of these genera infect >100 insect and crustacean species [7]. Patent infections involve massive levels of virus replication that result in infected larvae displaying marked iridescence, whereas covert infections may reduce the reproductive capacity of the host. The genera were previously distinguished based on virion size and the iridescent colour of infected larvae; phylogenetic analysis of complete genome sequences now gives a superior method of differentiation.

## Resources

Full ICTV Online (10th) Report: www.ictv.global/report/iridoviridae.
